# The opportunity to save a life: A qualitative study of a point-of-care overdose education and naloxone distribution intervention

**DOI:** 10.1371/journal.pone.0326495

**Published:** 2025-06-25

**Authors:** Janet A Parsons, Benjamin Markowitz, Rekha Thomas, Kris Norris, Mercy Charles, Chantelle King, Kate Sellen, Douglas M Campbell, Pamela Leece, Michelle Klaiman, Leigh Chapman, Shaun Hopkins, Rita Shahin, Curtis Handford, Vicky Stergiopoulos, Laurie J. Morrison, Carol Strike, Aaron M. Orkin, on behalf of the SOONER investigators

**Affiliations:** 1 Department of Occupational Science & Occupational Therapy, Temerty Faculty of Medicine, University of Toronto, Toronto, Ontario, Canada; 2 Li Ka Shing Knowledge Institute, St. Michael’s Hospital, Unity Health Toronto, Toronto, Ontario, Canada; 3 University of Limerick School of Medicine, Limerick, Ireland; 4 Applied Health Research Centre, St. Michael’s Hospital, Unity Health Toronto, Toronto, Ontario, Canada; 5 Health Design Studio, OCAD University, Toronto, Ontario, Canada; 6 Department of Pediatrics, St. Michael’s Hospital, Unity Health Toronto, Toronto, Ontario, Canada; 7 Public Health Ontario, Toronto, Ontario, Canada; 8 Dalla Lana School of Public Health, University of Toronto, Toronto, Ontario, Canada; 9 Department of Family and Community Medicine, Temerty Faculty of Medicine, University of Toronto, Toronto, Ontario, Canada; 10 Division of Emergency Medicine, Department of Medicine, University of Toronto, Toronto, Ontario, Canada; 11 Department of Emergency Medicine, St. Michael’s Hospital, Unity Health Toronto, Toronto, Ontario, Canada; 12 Lawrence S. Bloomberg Faculty of Nursing, University of Toronto, Toronto, Ontario, Canada; 13 The Works, Toronto Public Health, City of Toronto, Toronto, Ontario, Canada; 14 Toronto Public Health, City of Toronto, Toronto, Ontario, Canada; 15 Department of Family & Community Medicine, St. Michael’s Hospital, Unity Health Toronto, Toronto, Ontario, Canada; 16 Centre for Addiction and Mental Health, Toronto, Ontario, Canada; 17 Department of Psychiatry and Institute for Health Policy, Management and Evaluation, University of Toronto, Toronto, Ontario, Canada; 18 Department of Emergency Services, Sunnybrook Health Sciences Centre, Toronto, Ontario, Canada; 19 Department of Emergency Medicine, St Joseph’s Health Centre, Unity Health Toronto, Toronto, Ontario, Canada; University of New Mexico Health Sciences Center, UNITED STATES OF AMERICA

## Abstract

Canada’s opioid crisis continues to escalate. Naloxone can effectively reverse the effects of opioid overdose. We planned a randomized trial on the effectiveness of a point-of-care overdose education and naloxone distribution (OEND) intervention on participants’ performance in a simulated opioid overdose scenario. In preparation for the trial, we conducted a feasibility study which included a qualitative process evaluation aimed at eliciting participants’ perspectives of the study’s OEND tool and procedures, and how their lived experiences of the opioid crisis intersected with their experiences of the study. Twenty-three participants were interviewed, including people with lived experiences of opioid use or overdose, and people living in neighbourhoods or working in services where they were likely to encounter overdose. Thematic analysis of interview transcripts was informed by stigma theory. Participants’ accounts depicted challenges faced by people who take opioids in their everyday lives, deep losses experienced, negative attitudes encountered, and systemic barriers to care. Participation in the study itself was portrayed as meaningful. We explored participants’ experiences through three key themes: (1) *who were the participants –* describing their experiences related to opioid overdose, opioid use and attendant stigma; (2) *why did they participate –* recounting their motivations to join the study; and (3) *what they thought about study processes* – reflecting on the OEND materials and study procedures*.* Accounts revealed a sense of agency as participants confronted the opioid crisis. Our results demonstrate that people experiencing opioid use and overdose and people who care about them are eager *and* willing to be approached about research at point of care; participants were eager to learn overdose prevention skills and to return for follow-up study sessions. They recounted a range of motivations for participating, the most important of which is the opportunity to actively intervene, save lives and raise awareness. ***Trial Registration***: ClinicalTrials.gov registry (NCT03821649)

## Introduction/Background

Canada is in the midst of a prolonged epidemic of opioid-related deaths, with no signs of abating [1]. The number of lives lost and the pain and suffering endured by people who used opioids and their friends and loved ones, is staggering. Escalating drug toxicity, including a changing profile of adulterants such as benzodiazepines and other sedatives that cannot be reversed with naloxone [2,3], has made opioid overdose more difficult to reverse. Opioid-related stigma further compounds the crisis [1].

One positive development in recent years has been the increasing availability of naloxone, which can effectively reverse the effects of opioid overdose [4]. The advent of naloxone as a nasal spray offers an easy-to-use naloxone administration technology, and makes naloxone approachable to a much wider segment of the general population, including those who are not comfortable with needles or injection. This technology also prompts research on how to respond to an opioid overdose, incorporate naloxone distribution into general first aid education, and make naloxone distribution a part of routine clinical practice in settings where people who may witness opioid overdoses receive care [5].

We planned a randomized trial to test the effectiveness of a novel co-designed point-of-care overdose education and naloxone distribution (OEND) intervention on participants’ performance in a simulated opioid overdose scenario, in comparison with an existing community-based OEND program [5]. Randomized trials on naloxone distribution have faced numerous feasibility and analytical challenges [6]. Trials among people who use drugs and people who are likely to witness opioid overdose are often not pursued due to the real and perceived challenges in recruitment and retention. Simulation-based studies of OEND interventions are similarly rare due to the complexities of developing an acceptable and appropriate opioid overdose simulation. At the outset of our trial, it was therefore unclear whether eligible individuals would engage with the study, or how they would experience study participation.

This paper reports on a qualitative investigation, embedded in a feasibility study [7]. The goal of the feasibility study was to ascertain the feasibility of recruiting and retaining participants for a planned randomized control trial (RCT) [7]. Our qualitative investigation was designed to elicit participants’ perspectives of the study’s OEND tool and procedures, their experiences of recruitment and retention in the RCT, and how their lived experiences of the opioid crisis and responding to opioid overdose intersected with their experiences in the feasibility trial. This qualitative study was designed as a process evaluation to guide the successful engagement of people who are likely to witness opioid overdose in randomized trials and simulation studies.

Our study is informed by stigma theory, which is broad and complex and has evolved over time. We drew primarily on interactionist framings [8], which focus on the social relational aspects of stigma (as informed by Goffman, Scambler and others) [9–11], as well as critical and human rights framings of stigma [12], that attend to social structural aspects such as power, as well as systemic barriers, [10,12]. Because we were interested in how people who take opioids might feel when being invited into a research study, the relational approach to stigma was particularly informative as we were interested in how interactions with study personnel, with hospital/healthcare settings, and with study materials (such as the naloxone kit and overdose education materials we co-designed) [9,13]) might affect participants’ perceptions of our approach as sufficiently inclusive and respectful. We were also aware of structural aspects of stigma [8,12], and the ways in which experiences of opioid use/overdose intersect with issues of housing and income precarity, systemic barriers to care, as well as existing policies and laws. We attended to these complex and multi-faceted understandings of stigma as we designed our study.

## Materials and methods

### Study design

Qualitative interviews were conducted as part of the feasibility study, where study design and implementation, including details related to the design of our OEND intervention have been detailed elsewhere [5,7,9,13] with the aim of determining if and how people were willing to participate in a trial focused on OEND intervention, their willingness to participate as a responder in a mannequin-simulated opioid overdose, as well as a follow up interview.

As noted above, the qualitative study was informed by stigma theory [10,14]. The research team was keenly aware of stigma associated with taking opioids and with opioid overdose [15]. The Surviving Opioid Overdose with Naloxone Education and Resuscitation (SOONER) team included community members (people with lived experience of overdose/witnessing overdose, people who use drugs, and people providing harm reduction services in the community) and frontline healthcare practitioners (HCPs) who contributed to planning [9,13]. The core qualitative research team (JAP, BM, AO and CS) consisted of researchers with expertise in qualitative methodology (including community-based research), stigma, social theory, health equity, the opioid crisis and resuscitation science, while the interviewers (KN, RT and MC) had expertise in conducting research concerning sensitive topic areas, in-depth interviewing, and community-based approaches to research.

For this embedded qualitative investigation, we used an interpretivist approach to qualitative inquiry [16] to understand participants’ experiences of the study procedures and materials. As researchers, we were interested in how people make sense of their experiences (meaning making) [17], but also employed a critical theoretical stance [18] to the topic, in that we attended to the roles that power and structural forces play in the lives of people who take opioids.

### Study setting

The interviews took place at a single institution, an academic tertiary-care hospital located in downtown Toronto, Canada’s largest city. The study received ethical approval from the Research Ethics Boards of St. Michael’s Hospital (#17-280), University of Toronto (#RIS37212) and Toronto Public Health (#2017-09) [7].

### Sampling and recruitment

All participants were recruited for the feasibility study from one of four clinical services at St. Michael’s Hospital, specifically the Emergency Department, the Department of Family & Community Medicine, Addictions Medicine Services (inpatient and outpatient services) or the hospital-affiliated Inner City Family Health Team [7]. Eligibility criteria included being at least 16 years old and being at increased likelihood to witness or experience opioid overdose. Both patients at one of the recruiting sites or persons accompanying a patient were eligible to participate [7]. Recruitment began on 01/02/2019 and recruitment ended on 29/08/2019. Interviews happened a few days after recruitment to the study. Participants gave verbal informed consent and the consent was documented and witnessed by the research coordinators. Aided by an outline of study procedures and a brief script, ample time was allotted for participants to ask questions and discuss each phase of the study [5]. Participants received $75 CAD total in cash for completing the study procedures, as well as transit tokens and refreshments [7].

### Data collection

The feasibility study procedures for data collection and the simulation assessment procedures have been described elsewhere [7,19]. Here we review a few key aspects, relevant to our qualitative investigation. Upon recruitment participants were randomly assigned to one of two groups – experimental and control. Those in the experimental group received a “purpose-designed point-of-care brief overdose first aid training and a naloxone kit, containing two doses of intranasal naloxone (Nar-canTM, Adapt Pharmaceuticals) with administration instructions” [7, p.3]. This kit and associated training materials (animation) were co-designed through an iterative process described elsewhere [9,13]. Those in the control arm received best available care including referral to existing local OEND programs at either a local pharmacy or harm reduction program [7]. All participants, regardless of which arm of the trial they were enrolled in, were invited back to participate in a high-fidelity opioid overdose simulation 3–14 days following recruitment [7]. Immediately following the simulation, the participants were debriefed in a structured format [19]. Design and development of the simulation assessments, the results related to simulation-specific debriefing interviews, and participants’ in-depth reflections on the simulation itself are reported in detail elsewhere [19], and are not the focus of the present manuscript.

All participants were interviewed immediately following the simulation debriefing. This set of interviews (the focus of this paper) had a different purpose; using a semi-structured interview guide, topics covered included their experiences related to taking opioids, experiences of witnessing overdose, and experiences of the study materials and procedures. All interviews were conducted by experienced research coordinators (KN, MC, and RT) responsible for recruiting participants and implementing the protocol. All interviews were digitally audio recorded.

### Data analysis

The audio recordings of the interviews were transcribed verbatim and checked for accuracy. We conducted a thematic analysis (TA) of the interview transcripts, modeling our approach after Braun & Clarke’s reflexive TA [20,21], but using elements of a codebook approach from King and Brooks’ (2017) template analysis [22]. As noted by Braun et al (2019), this interpretive approach is sufficiently flexible and allows for the incorporation of theory [20]. Steps entailed initially reading through the transcripts and familiarizing ourselves with the data and then generating initial codes, using a combination of inductive and deductive orientations [20,22,23]. An initial coding framework was developed and then refined based on team discussions [22]. We constructed themes, first prototyping them to determine if they helped organize the data in a meaningful way [20], and then proceeding to define, revise and refine the themes through our ongoing analysis and team discussions [22]. Finally, we revisited the central organizing concept for this project, which related to understanding if it was possible to recruit and retain participants experienced in opioid taking or overdose into an RCT, whereby we developed the overarching architecture for our analysis (final thematic structure): *who* participated, *why* they participated, and *what* their experiences of participation were like. A copy of the coding framework appears as a supplementary file. While our approach was largely inductive, deductive coding entailed attending to aspects of the text that reflected key features of interest *a priori*, such as those related to experiences of study procedures (process evaluation) and stigma.

The interview analysis was led by team members experienced in qualitative methods (JAP, BM). Coding of the first 9 transcripts was completed by BM, who then reviewed and discussed the developing coding framework with a senior qualitative researcher (JAP). BM and JAP then met with the senior authors (AO and CS – also highly experienced qualitative researchers and content experts and the interviewers (KN, RT and MC)) to discuss the developing coding framework and to deepen the interpretation of these preliminary results. BM and JAP then worked on developing and refining the initial themes, for application across the data corpus, bringing these back to meetings of the core team for further discussion and refinement.

Our interpretation was informed by stigma theory (as noted above), specifically theories that focus on relational aspects including enacted and internalized stigma, as well as issues of power and discrimination, and critical perspectives (which attend to structural stigma) [8,10]. For example, we attended to participants’ accounts where they spoke about how they were treated when seeking healthcare, where opioid use intersected with experiences of housing precarity, or where they experienced barriers to accessing mental health and addictions services. As the analysis progressed, we also reflected on concepts of agency [24–26] and counter-story [27,28].

## Results/Findings

A total of 23 people participated in the interviews, with 7 participants self-identified as women, 14 self-identified as men, and 2 self-identified as two-spirited and queer. Participants ranged in age between 27 years and 67 years (median = 43). Simulation sessions and interviews were conducted between February 2019 and September 2019. Qualitative interviews typically were between 45 and 60 minutes duration. Twelve participants identified as a person who takes opioids, while 11 said they cared about someone who takes opioids (e.g., friends, family).

The interviews paint a complex portrait of the challenges faced by people who take opioids in their everyday lives, the deep losses experienced, negative attitudes encountered, and systemic barriers to care. Participants also spoke passionately of their desire to be able to help others (e.g., friends, family, neighbours, fellow citizens), offered advice on how we might improve our intervention, and how care and support could be improved more broadly for people who take opioids. Their accounts spoke to a sense of *agency* that accompanied acquiring skills to confront the opioid crisis in their communities first hand. Participation in the study was portrayed as a positive opportunity for social contribution in the context of a health crisis and personal loss. As we reflected on our developing analysis, we recognized that their approach to participation was itself an important part of the story, and it is depicted through three key themes: *who were the participants*, *why did they participate*, and *what did they think about the study processes.*

### Who were the participants?

Beyond the ‘standard’ description of participants offered above, it is meaningful to understand the lived experiences and perspectives that the participants brought with them into the study space. In order to understand their reflections upon the study processes, materials, and their experiences of study participation, the broader contexts and circumstances in which participants were living their lives becomes particularly salient. Participants gave complex and in-depth accounts of their experiences related to taking opioids and opioid overdose (either their own or someone they cared about). In addition, an ever-present ‘character’ in their stories was the stigma related to opioid use. All of these experiences not only informed their interest in the study, but also their perspectives on overdose education and better accessibility to naloxone

Participants included people who had lived experiences of opioid use and overdose (either themselves or friends and family members), people who lived in neighbourhoods where they were likely to encounter someone experiencing opioid overdose, and people who worked in services where they might encounter opioid overdose (e.g., social service agencies, sex work). They spoke to multiple sources of inequity they had experienced. Poverty, homelessness, child custody loss, and involvement with the criminal justice system were some of the marginalizing circumstances they experienced which intersected with their own experiences of opioid use or those of others. These difficult social circumstances were characterized as predisposing one to encountering or experiencing opioid overdose oneself.

The participants recounted their *experiences related to opioid overdose, opioid use* and *attendant stigma*.

### Experiences of opioid overdose

Participants told of witnessing opioid overdose, fears they had for themselves and others regarding opioid overdose, and concerns that they had about how it was being addressed at the time of the study (which pre-dated the pandemic). These included firsthand accounts of what it was like to have opioid overdose; what it was like to have people help them during an overdose (professional or lay); and what it was like to witness an opioid overdose. A participant commented,

“I have a long drug history, I’ve been on the streets for a while. I, I’ve seen a lot of drug related overdoses and yeah. I’ve been around when people were getting revived from an overdose. It’s not a fun thing to watch, especially when people kind of react in panic and confusion.” (FS-P31)

Participants often framed their accounts in terms of their concerns for others with similar experiences.

“.. you can see someone in the subway just dropping and I, I seen it, when someone drops down you know, like face planting on the subway, nobody is helping, you know. I tried to help him to the best of my ability, right? But yeah, there’s some people they either get scared or they don’t care.” (FS-P9)

References to fear were common: opioid overdose was characterized as scary, unpredictable, and “messy” for the person experiencing opioid overdose and the people around them. The participant’s comment that “nobody is helping” – and their assertion that this might be because nobody cares – imparts a sense of frustration and isolation. The speaker offers a counter-story that they actually *do* care, which informed their decision to be part of the study.

### Experiences of opioid use

The majority of participants had long and complex histories related to opioid use. Some said they were currently taking opioids, some described a range of rehabilitation and detox interventions. They depicted a range of pathways into opioid use – for example, through prescription medications, or being introduced to taking various drugs (non-medically) through partners and friends. Some spoke about starting on opioids first, while others described a more gradual journey into taking increasingly ‘hard’ substances. Some participants recounted having concomitant health conditions which intersected with their opioid taking, including mental health concerns (e.g., anxiety, depression), injuries, chronic pain and cancer. One of the participants recounted being diagnosed with cancer years previously, and how their taking of prescribed opioids evolved into dependence,

“I thought okay, this is a pill, I will take this pill as prescribed, and the next day if I didn’t feel I needed it, then I, everything would be fine. That’s what I was thinking. Boy, was I wrong, wow! I was wrong … there would be times where I couldn’t get out of bed, I couldn’t eat, couldn’t even hold down a glass of water, you know, it’s actually so gripping that you feel it’s killing you.” (FS-P10)

Others told of experimenting with drugs during their teens; that they started out simply being curious, while one participant told of being given opioids unwittingly, saying “The first time I did opioids that was actually not my choice, somebody injected me with, with heroin…” (FS-P14).

Participants also talked about experiences with a range of different opioids and an increasingly dangerous and toxic drug supply. Some participants noted that the advent of carfentanil/fentanyl analogues was making drug use more risky than previously. Participants exhibited a strong awareness of these risks, recounted their strategies for navigating them, and expressed interest in learning about overdose first aid and resuscitation in this evolving context.

### Experiences of attendant stigma

Comments regarding stigma and reflections on prevailing societal discourses surrounding opioid use/overdose represented a recurring theme throughout the dataset. Stigma was portrayed as deepening the social isolation felt by those struggling with addiction. The damaging effects of stigma were highlighted.

Participants told of encountering situations in which they felt judged or mistreated due to their history of opioid use. While this could happen in their daily lives, it also occurred when they sought healthcare. Participants recounted negative attitudes from healthcare providers, which could impact their willingness to seek emergency care, even in cases of opioid overdose. As one participant stated, “me personally, I don’t like going to the hospital when I OD.” (FS-P8). That stigma can be a barrier to life-saving care speaks to its power to shape people’s experiences and perspectives and its potential to impact health outcomes.

Another participant commented,

“You’re going to have the doctor, the physician actually judge, be judgmental to you … Plus being homeless, that kind of people think, these people are expendable, when they are not, you know.“(FS-P10)

Here, the participant provides important insights on intersecting sources of inequity and stigmatizing attitudes, but they also push back against (resist) such negative discourses, countering “they are not, you know.” Other participants also offered counter-narratives and addressed prevailing discourses very directly. One participant commented,

“There’s a stigma, ‘cause everyone thinks that if you’re on opioids… there’s something wrong with you. Even when you take medication for anxiety or depression there’s something wrong with you…. It’s like someone who is diabetic; they need their insulin, otherwise they’re going to be running around the world impaired. Well, somebody who’s on opioids… they are doing the best they can with what they got. And they’re in pain or they are suffering and a doctor has told them [to] go on opioids. They’re not out there being like ’I’m on opioids because I decided to.’ Someone has put them on opioids and then from there, addiction has occurred.” (FS-P33)

Given their prior experiences and the fact that our study was situated in a hospital and led by healthcare professional researchers, it was all the more remarkable that participants were eager to take part. We identified a narrative tension in some of the interviews, in that people talked both positively and negatively of their experiences in seeking health care. While the above accounts of stigma are disturbing, there were often expressions of gratitude for the study and how it was perceived as a well-designed vehicle to genuinely help people at risk of overdose.

### Why did they participate?

“I just want to try to help if I can” (FS-P22)

Participants reflected on what motivated them to participate in this study and they had a number of reasons for doing so. Some explained it in terms of their own experiences of taking opioids (as well as overdose) or those they cared about. Many of them spoke about the desire to protect people in their community from overdosing and to save a life if they could. A participant commented that they wanted “just to learn how to save somebody’s life, simple as that.” (FS-P1), reflecting a motivation common to most.

“Seeing my friends suffer and not having a way out and seeing that pattern become so often and so cyclical being like opioid, addiction, street drug, depression increases, addiction increases, and even suicidal thoughts and self-harm increases. I see this all the time and it’s really challenging for me and only me to deal with it. So, seeing that study I was like yup, let’s– let’s do this. And let’s have a conversation.” (FS-P33)

This desire to help the people they knew was mentioned repeatedly, conveying a sense of their highly personal interests at stake, a feeling of agency, and placing value in being prepared to act. The idea of “being prepared” to intervene actively on behalf of those they cared for was expressed often. Some participants were approached for the study when they had accompanied someone they cared for to the emergency department. At first, some said they assumed that they would be asked to complete a survey, but were pleasantly surprised to know that they would learn about overdose prevention and this excited them.

A number of people mentioned that the study’s cash honorarium was also a welcome aspect that helped to motivate them further, with some participants making reference to experiencing low income. However, they were quick to qualify this, by expressing their desire to help others as well.

“… it’s not about the reward either you know, because I, I, I yeah, I do need the money but it’s more the educational part of it, right? Especially because I am an addict you know, and I am surrounded by addicts so it’s, it’s helpful.” (FS-P9)

This idea of being ‘surrounded by overdose’, meaning that they reported witnessing overdoses repeatedly, was echoed by numerous participants.

“I don’t think people realize how like, how people are dying … it’s happening every day. So, you can go to the [shelter] right there and watch somebody drop right? So, it’s, it’s good that, what you guys are doing. So I think it’s just a thing, a humanitarian thing to do.” (FS-P7)

Others commented on the nature and context of their work as a consideration when deciding to participate in the study. As one person commented,

“…being exposed to that scene, like the sex work scene, there’s a lot of drug use that’s become super normalized, so it’s good to know how to react, if there’s an overdose.” (FS-P16)

Another participant said the study piqued their interest because of working in a non-profit organization that supports clients who might be at risk of overdose. They expressed surprise that they had never been offered overdose education by their managers, and wanted to participate because they had never done overdose response training before.

But in some cases, participants said that they simply joined the study when their physicians mentioned it. Still others said that the study sounded interesting and they wanted to try something new.

“I think that I done it because I never had a chance to do it. You know, it was, it was kind of interesting. I think the whole study is interesting to be honest with you…” (FS-P6)

The people who volunteered for our study were not necessarily people who would have had opportunities to engage with first aid training previously. For many of them, the idea that they could learn “to save a life” seemed new to them, and was embraced with enthusiasm and curiosity. This depiction of what prompted participants to volunteer for the study indicates that this was very much linked to who they were as participants, in terms of their experiences related to opioid overdose, opioid use, and opioid-related stigma. It also informed their responses to study processes and materials.

### What did they think about the study processes and materials?

We conducted the qualitative interviews with the intent of refining our study processes, understanding barriers to participation encountered, as well as getting a sense of who was volunteering for our study and why. We asked participants to tell us about what it was like to engage with our study, including processes of recruitment, scheduling, receiving the point-of-care OEND, and navigating the simulation appointment. Participants offered insights into how we might improve elements of our educational intervention (animation and kit), things we could do to improve our procedures, and how the simulation might be enhanced.

As mentioned at the outset of this paper, one of our goals was to make our intervention user-friendly, practical and memorable; but we also wanted to ensure that the intervention and our study procedures contributed to reducing stigma. As we have seen, participants spoke of multiple sources of marginalization that people who take opioids experience. We knew that these represented real barriers to care, including the willingness of bystanders to assist with overdose resuscitation. It was important for us to know whether we had succeeded in creating an intervention and study processes that were acceptable, supportive and practically useful to people most in need of support in reversing opioid overdose.

### Experiences of study procedures

Participants were asked to reflect on how they were approached about the study, and about the OEND intervention offered. Overall, participants appreciated the way that study materials and processes were set up, and felt that study personnel treated them well. Participants’ comments about the study itself were overwhelmingly positive – and even when they had suggestions for improvement, they were quick to emphasize how much they appreciated the study, its topic, and a chance to learn something practical so that they “could save a life.”

People commented that their first interaction with the study was either taking an information card that had the study information on it, or being told about it by their physician who thought they might be interested in it. A participant suggested that the card introducing the study could be reworded:

“The only recommendation … I would make on the card itself is the fact that the very first line states ‘do you use opioids?”’ and the second line states ‘does somebody you care about use opioids?’ And the fact is, if you are the opioid user chances are you are not going to be giving yourself CPR or naloxone so it should be ‘do you care about somebody that uses?’ and then you know, ‘do you use yourself’?” (FS-P3)

Participants commented that they felt the study coordinators explained the study and its aims well. Participants frequently remarked that they felt the coordinators supported them and were helpful and kind. Once participants had been recruited and had consented to the study, they were randomized to the experimental or control arm and provided the intervention based on their allocation. They were told about the simulation appointment and scheduled to return to the hospital’s simulation centre. They received multiple reminders by phone and text message from the study coordinators regarding their upcoming simulation session [5]. Most said they appreciated the frequent reminders stating, “it reminded me because I was thinking that uh, I didn’t realize it was today when you called yesterday.” (FS-P1).

There were a few participants who thought the reminders seemed a little too frequent. As one participant said, “I think overall it was just, it was a little bit much for me the reminders but I think, it’s not, it’s not that big of a problem, like it’s just a tiny annoyance.” (FS-P15)

More than one participant had reported being treated in negative ways from some staff who worked at the study hospital (not study personnel). One participant commented at length about coming in to the hospital to meet the coordinators and being treated judgmentally by hospital security staff. She noted that

“But when I came in [to the lobby] the security was kind of looking at me like ‘what’s this homeless girl,’ like I just felt judgemental from him…. I said ‘is there a [coffee shop name] in [here]?’ At first he told me ‘no.’ And then I pulled out your card and then his tune switched up and he’s like ‘yeah, it’s, it’s right there’. … for me to, to have to show a card, to let them know that I am here for a reason is not, is not fair. It’s not fair at all.” (FS-P8)

This demonstrates that, despite a team of investigators working across disciplines and with commitment to designing a non-stigmatizing intervention and study processes, negative interactions and stigmatizing behaviour could still occur, despite best efforts. This reflects the difficult interactions people affected by opioid overdose encounter on a daily basis and from multiple sources. We interpreted the willingness of participants to persist with the study despite such experiences as a high level of commitment to contributing to overdose resuscitation research. This particular event also led to refinements in study processes and an educational outreach with security staff to engage them with the project.

While we do not report specifically on findings related to participants’ simulation experiences here (this is the focus of another paper) [19], it is important to note that participants offered in-depth accounts of how supportive and engaging the simulation experience was. Participants said they appreciated the opportunity to practice resuscitation skills in real time and receive feedback on their performance, that it was a practical learning opportunity [19]. Some participants offered suggestions for making the simulation experience more realistic (saying the apartment looked too clean, or that in real life it might not always be as obvious that opioids had been taken). One participant suggested that the simulation experience itself could serve as a basic-level course in overdose resuscitation and that it could be built upon for “progressive overload” (FS-P33), whereby resuscitation scenarios become increasingly complex, with more distractions added in,

“Once they get ten out of ten times perfect now, [then] make it harder. You’re gonna make it weirder. You’re gonna have music on in the background. They gotta think while music’s going on […] Now it’s like an escape room, you have to think and solve problems. You have to solve problems while this guy’s life is on the line.” (FS-P33]

### Perspectives on the animation

For participants in the intervention group (n = 16), reactions to the educational animation varied. Some participants had trouble remembering seeing the animation when they were first introduced to it. Others really liked it and said they remembered it well. A participant commented that it was pretty self-explanatory, when accompanied by the kit,

“…you know, with a 5-minute training, I mean realistically, it’s a video [animation] and as you have demonstrated you know, it’s very simple. You open it up and it says call 911, it says you know, it’s, the packaging is very explanatory, you know, shake, shout, call 911…” (FS-P1)

Participants were asked to reflect on the use of animation in the training.

“… they weren’t like completely human-like and I liked that. I liked that…I am super like, hyper critical of things like that because [of the ways] that certain communities or like races, are portrayed in, in certain videos [animations] is going to be like super problematic. And having a human-like figure that isn’t like completely specific to any race or a sexual preference made me feel more comfortable to watch it. And I guess a little more focus[ed] on the actual information that’s being given.” (FS-P16)

This participant suggests that using less specifically humanoid characters was a benefit because it allowed them to focus more on the content/information being conveyed, rather than being distracted about ‘who’ was being portrayed or not portrayed in the animation. This suggests that the use of animation made it less likely to attribute certain characteristics to persons who might be taking opioids or responding to opioid overdose.

However, other participants felt differently. A few participants said that the use of animation, and the way the characters and situation were portrayed made it “seem foolish” for such a serious topic. “The terms, of the tone and the clarity were fantastic. It was just the imagery seemed a little foolish in, in comparison to the actual problem.” (FS-P3) This person went on to say, “Other than the really bad graphics it was perfectly fine.” (FS-P3) Another participant commented that they would have preferred a live action video rather than an animation.

“It would be much better seeing somebody actually doing it, you know…so if 10 people did not know how to use naloxone and they were watching that [animation] and watching a live [video], I think … 10 out of 10 would be saved watching the live [action] video and maybe 7 out of 10 would be saved watching the [animated]. It’s just people should have a more, you should watch both but if you are going to put one video out there I think it should be live rather than [animated] So, that’s me personally. Some people are different visually.” (FS-P7)

Despite the differences in reactions to the training’s format and design, most participants said that the animation helped them to understand the steps involved in resuscitation, including the need for chest compressions and contacting EMS.

“I think it made clear the important steps and like the real important ones … calling 911, and chest compressions and giving them naloxone and giving it to them again after 5 minutes if they are not breathing. I think it was really good at like giving/getting those important things to stand out.” (FS-P15)

Even those who said that they had difficulty recalling what they had seen with their first exposure to the animation, found participating in the simulation consolidated their learning and made the steps clearer.

### Carrying the kit

Participants remarked on features of the kit that had been designed specifically as part of the intervention [9,13], and which was provided to them upon entering the study (which can be seen at www.soonerproject.ca). It contained two doses of naloxone as well as a pamphlet outlining steps in resuscitation for overdose. At the time of the feasibility study, nasally administered naloxone was less familiar to some participants, with some expressing being more comfortable with the injectable form. Most people liked the fact that the kit was both discrete but easily recognizable as something ‘medical’ – while not being entirely obvious that it had to do with naloxone.

“I like the green packaging, meaning it’s actually, it actually seems to be, the green sort of snake, serpent and for me it means medical or emergency and therefore I am far more proud to carry it. And it doesn’t pop out as naloxone, when I am in a coffee shop … I’d hook this onto my backpack. But the previous naloxone kit [they had used], it makes you look like an addict, a user, and I have had reactions to me carrying a naloxone kit.” (FS-P30)

This participant gave a long and detailed account about the stigma associated with taking opioids, with carrying naloxone, being identified as someone who takes opioids, and the concerns they had about this silencing important conversations that need to happen regarding the opioid crisis. Their reflections on the kit were closely interwoven with their perspectives on what needs to change in terms of attitudes towards opioid overdose more broadly. Like some of the other participants, this participant took an activist stance, pushing back against silences and stigma that push overdose away from view and care.

Some participants said that they would like the kit to be more clearly marked to show what it is for.

“Maybe it [the kit] should have something identifying much more on the side - like this doesn’t really, what does this identify? So, I don’t know, just looking at it, I had to look at it for a second. It’s obviously a kit, there’s nothing else here, but looking at this—like is that a health plus sign? It’s not the, typically the colour red or blue, red cross you know, so that’s all I can say about that.” (FS-P7)

Many participants said that they would carry the kit with them, and were interested in offering naloxone dosages to their friends and family members. Because it “wasn’t big and bulky”, the experimental kit was easy to carry, although some said the need to include two naloxone dosages made it a bit bigger than they would like. A few people said that they were unlikely to carry it, but instead planned to keep it at home like they would a first aid kit.

In terms of expressed motivations for carrying the kit, many participants said that they would carry it so that they could intervene and help someone else, which aligned with their motivations for learning about overdose resuscitation. Some people also said that they would carry it with them in case they themselves overdosed and hoped that someone would search them for naloxone, find it and administer it. As one participant commented, “I believe every person that’s using [opioids] that they should have a kit.”(FS-P14).

Overall, our participants responded favourably to the study’s processes and materials, and the way in which research personnel made them feel welcome and supported. They also had constructive feedback for us that is important to further refining our point-of-care intervention.

## Discussion

When we began the study, we were uncertain whether our recruitment processes would prove effective, whether stigmatizing discourses surrounding opioid use and opioid overdose might prevent people from participating, or whether people would even be interested in learning about resuscitation for opioid overdose. We were able to demonstrate that people *are* willing to be approached at point of care and that they have a range of motivations for participating, the most important of which is the opportunity “to save a life.” We were also able to demonstrate that people regularly taking opioids were very willing to return for follow-up sessions (e.g., simulation assessments, interviews), were eager to learn overdose prevention skills, and generously shared their stories and insights with the study coordinators.

Overall, participants displayed a sense of agency, that they had the potential to be ‘change agents’ within the opioid crisis. “To be an agent is to intentionally make things happen by one’s actions” [24, p.2]. Personal agency implies intentionality, forethought, self-regulation and self-reflectiveness [24]. Participants expressed genuine interest in the educational intervention and showed enthusiasm for the simulation and the experiential learning, including opportunities to provide feedback. Together, this suggests that people who take opioids and those who care about people who take opioids would appreciate further opportunities to consolidate their skills. This aligns with conceptualizations of agency whereby engagement is informed by the past (prior experiences), while grounded in the present (attending to current circumstances) and oriented to the future (“imagin(ing) alternative possibilities”) [29, p.962]. However, such individualized views of agency ignore important issues of “practice”, whereby action is situated and influenced by the immediate environment [26]. The idea of offering in-person overdose resuscitation courses (like a Basic Life Support course) in the community with those most at risk of overdose is an exciting possibility. However, our study demonstrates that such courses would need to be designed with care and sensitivity, so that learners would not feel further stigmatized and so that the opportunity to build agency through these offerings is realized. As we have shown, stigmatizing experiences can still occur even in studies such as ours, which has been designed throughout to address community members’ needs and concerns. As we have shown, a study intentionally designed to address the needs and concerns of community members can facilitate positive and affirming experiences in research, however, it does not completely eliminate the risk of stigmatizing experiences, as the study is situated in the broader social context that produces and perpetuates such stigma. Thus, agency is not just bounded by the individual, but needs to account for social structures that continue to marginalize people who take opioids, making the need for inclusive study and service design all the more crucial. For example, in our investigation, participants offered important counter-narratives [27,28] to stigmatizing discourses surrounding opioid use; they portrayed themselves as committed to saving lives and as agents of change (rather than as passive ‘victims’ of overdose); they offered important and insightful feedback on how to improve the intervention as well as its evaluation; they gave feedback on study materials such as the naloxone kit, including when and how they would carry it; they demonstrated a high degree of engagement as participants, counteracting assumptions that people who take opioids would not return for multiple visits as part of a study.

Participants brought a range of perspectives to the study. Over half were people with lived experiences of taking opioids and who had witnessed overdoses, with some friends and service workers also participating. Their complex accounts of experiences of opioid use, of opioid overdose, of stigma and discrimination, of grief and loss, and of multiple sources of inequity all informed and intersected with their experiences of our OEND intervention; these accounts were very similar across groups (i.e., whether a participant identified as a person who took opioids, someone who cared about persons taking opioids, or someone likely to encounter opioid overdose). Being treated with respect throughout the study was valued by participants – and we would argue this was an essential ingredient to participant retention. In general, people commented that they felt well-treated by study staff, did not perceive the intervention components as stigmatizing, and were able to offer suggestions for improving these components. As far back as Goffman (1963) [10], agency has been recognized as situated and relational [26]. Agency occurs in a context, in a setting (in our case, a study setting) which is influenced by the surrounding social and material circumstances [26] – both within and outside the study context. This is important, as the study procedures and materials served to encourage agency amongst our participants.

The population health effects of OEND programs are now widely known: increasing access to naloxone and associated overdose first aid skills reduces opioid overdose deaths [30]. With this evidentiary substrate, OEND programs have evolved from community activism, to niche services delivered in specialized substance use programs, to a mainstay harm reduction intervention and key component in the public health response to the opioid overdose crisis. Umbrella reviews of systematic reviews on OEND programs also demonstrate that more work is needed to develop and optimize OEND program delivery, including the details of training, naloxone kit design, and program adaptation to a variety of service delivery settings [30]. Our study contributes to this literature and need with a focus on service user experiences of opioid-associated stigma, naloxone kit design, and training tools.

Our study also contributes to threads in the literature about the challenges of research recruitment and retention among people who use drugs and other marginalized populations. These challenges are conventionally conceptualized as they relate to mistrust and distrust, where marginalized communities avoid research or choose not to participate because of deficiencies in trust between affected communities and health researchers [31]. The resulting research focuses on strategies to build trust and incentivize participation, and our study procedures certainly drew lessons from this research by offering cash incentives and co-designing study processes to be as accommodating and respectful as possible [5,32]. Our study findings add to this literature by demonstrating that research can also affirm participant agency, reduce stigma, offer rewarding and meaningful experiences for participants, and reinforce participant strengths. Our findings demonstrate that research need not be seen as an extractive process or one that places undue demands on marginalized populations: it can instead serve as a vehicle to affirm and advance the resilience of those communities.

This study has limitations. We are only able to report these qualitative results as part of our feasibility study, which was conducted in preparation for a randomized trial – a trial that was not able to proceed because of the advent of the COVID-19 pandemic. Nevertheless, our participants were able to offer in-depth accounts of their experiences of the study processes. The pandemic has only served to deepen the opioid overdose crisis occurring in Canada and elsewhere, and pushed more people into situations where overdose was often un-witnessed and with no one present to save a life [1]. Although the pandemic resulted in an unexpected interruption to our study, it also reinforced the need for novel, virtual, and asynchronous OEND interventions that are less reliant on in-person interactions to access lifesaving naloxone, which was the focus of our study. Another limitation is that this was a single-centre study, though it was conducted at a large urban teaching hospital that sees a large number of people experiencing overdose annually. While qualitative research is not meant to be generalizable at the population level, the themes generated appear broadly transferable across a range of settings, and our analysis was conducted to elicit generalizable concepts rather than represent all possible perspectives.

The complexity of conducting research in a field that is constantly changing should not be underestimated. Overdose OEND and resuscitation interventions continue to evolve, as do the ways in which people are able to access naloxone. In addition, the toxicity profiles of opioids (and other drugs) circulating among the Canadian population keep increasing, making overdose more challenging to reverse. It is crucial to design study procedures that are sensitive to the range of lived experiences that inform study participation. Many participants were continuing to struggle with opioid use, grief and loss, housing and food insecurity, disrupted relationships, stigma and discrimination. It was complex to conduct interviews with people who had just completed a simulation experience – one which some participants found demanding and emotionally charged [19]. It is important to build in personnel and supportive services that participants can avail themselves of if they need to.

Our study prompts numerous questions and opportunities for future research. OEND tools, kits, and educational processes can still be refined and adapted to make them more accessible, adaptable, and approachable across a variety of in-person and virtual service delivery settings, providers, and clients. The SOONER kit and training tools provide an initial platform and lessons for future innovation and adaptation. Our research points to future research directions across a range of studies that engage people who use drugs more consistently as partners in research, under the premise that research participation can be a positive and affirming experience rather than an extractive or distrustful one. Future research might explore what factors enable and sustain these positive experiences and how they might be used to transform ongoing engagement with research among people who use drugs and other marginalized or stigmatized communities. While we conducted our study with people who were seeking care within hospital settings, some participants noted a reluctance to seek health care because of fear of being stigmatized. It would be interesting to conduct a similar study but outside of hospital settings to compare results.

Our study demonstrates that people experiencing opioid use and overdose and the people who care about them are eager *and* willing to engage not only in research but in overdose training. The ability to actively intervene, to save lives, and to raise awareness were highly valued by our participants. A participant expressed their hope that the study would illuminate the significant challenges faced by people living through the opioid crisis, commenting,

“…like you can reach more people right? And just make them aware of, of these types of situations right? … there’s always that, that little hope that maybe someone can listen or someone can learn how to do this type of stuff in order to save a life. It doesn’t matter who it is, a life is a life.” (FS-P9)

## Supporting information

S1 File Coding Framework. (DOCX)
