# Supplementary material for: The opportunity to save a life: A qualitative study of a point-of-care overdose education and naloxone distribution intervention
Source: PLoS One. 2025 Jun 25;20(6):e0326495. doi: 10.1371/journal.pone.0326495 (PMC12192056; doi:10.1371/journal.pone.0326495)
Supplement: S1 File — (DOCX) [file pone.0326495.s001.docx]

**Supplementary File: Coding Framework**

| **Overarching/**  **Organizing Themes** | **Code names** | **Code Definition** | **Example quotes** |
| --- | --- | --- | --- |
| Who were the participants | Experiences of opioid use | Experiences of *taking* opioids, how they *began* using opioids themselves or seeing those around them take opioids  *Includes:* circumstances surrounding/initiating opioid taking; experiences with a range of opioids (different agents, other drugs); interactions with people who are using opioids  Links with: ‘co-morbidities’  (related and unrelated to opioid use)  Links also with: experiences of recovery and ‘detox’ | FS-P6: “The [currently circulating] drugs are a whole new level. They’re stronger, there’s bigger risks with them, a lot more man-made drugs here, so you never know when is your last hit.”  FS-P1: “… I was in my late teens and I started off doing Percocet and dealing with pills and it progressed to you know, doing other pharmaceutical opioids and then, I was doing heroin, just experimenting with it.” |
|  | Experiences of opioid overdose | Accounts of overdosing themselves or witnessing others overdose  *Includes:* first-hand accounts of what it is like to have an overdose; what it was like to have people help them (professional or community member); what it is like to witness overdose; fear; noticing opportunities to help | FS-P31: “ I have a long drug history, I’ve been on the streets for a while. I’ve seen a lot of drug-related overdoses and yeah, I’ve been around when people were getting revived from an overdose. It’s not a fun thing to watch, especially when people kind of react in panic and confusion.”  FS-P7: “… usually when I am using I am with the same group of people, so I am not going to say where, but you know, there’s ODs there every single day.So normally it’s common to actually find somebody …”  FS-P8: “Right now it’s all carfentanil, so I told [my friend] to split her dose in two rigs and she is like ‘no, I got this and I am okay’, and I am like ‘please split it in two’ and she didn’t. And we were in the washroom and [at first] she was okay, and then as soon as we got into our room she went stiff and blue, and I had to give her the naloxone and I got staff involved and, and yeah, they called 911.” |
|  | Stigma | Negative attitudes of others (enacted stigma), attitudes towards self (internalized stigma)  *Includes:* Situations where they felt misjudged or mistreated; Discourses surrounding opioid taking and opioid overdose  *Intersects* with other sources of inequity: e.g. housing precarity, criminal justice involvement, etc. Also intersects with experiences of discrimination and with relationships with family & friends. Links to counter-narratives | FS-P15: “…when people think about people who are addicts they, they tend to have a very rigid idea.”  FS-P9: “I was lucky that my family tried to help me, and tried to do something about it rather than expel me out of their life right? Some people do that and I have seen it like, ‘you’re a drug addict so get out of here, right?’  FS-P8: “…when I came in the security guard was kind of looking at me like ‘what’s this homeless [person] doing here?’ Like I just felt judgmental from him.”  FS-P1: “I deserved it [withdrawals in jail] … it’s alright, I put myself in these positions, right?”  FS-33: “You may tell your friends … that you’re sober cause you want some sort of praise or recognition for good behavior even though it’s not real, but you want someone to think it’s real. You may even convince yourself some days that you are sober, and you are a good person because you want some sort of validation...” |
|  |  |  |  |
| Why did they participate | Motivations to participate | What made an individual want to join the study; why they decided to join the study  *Includes:* An opportunity to learn overdose resuscitation (to save a life); discussion about existing knowledge regarding overdose response and desire to ‘learn what to do’  Also links to the idea of supporting one’s peers, and of activism (e.g., feeling connected to harm reduction community) | FS-P1: “in order to save somebody’s life properly with the naloxone kit, and then, yeah, just to learn how to save somebody’s life, simple as that.”  FS-P14: “What helps me is getting involved with harm reduction [community]…”  FS-P9: I want to share my experience [with] other people right, because [opioid use] takes over the life of people that they have addictions right? And sometimes we don’t know that there is ways to help others,, and there’s solutions that can be applied to change life and so I, I like studies [like this].” |
|  | Family/friends | Relational aspects of participants’ lives; motivating factor in participating because of consequences related to family and friends (e.g. deaths of family/friends, child custody loss)  *Includes:* relationships can promote or inhibit opioid use | FS-P10: “I would love to see if I know how to, how to resuscitate a friend, just in case they get sick you know?”  FS-P9: “…it’s a reality you know, and I, I seen it in … some of my family have died because of their addiction and I see a lot of friends dying over this.” |
|  | Living situation | Comments about living circumstances; how that links to their need for overdose response training.  *Includes:* living alone or in shelters (do they perceive themselves or others at risk of overdose); are there trained people around to help; region they live in | FS-P3: “I live in a basement apartment by myself.”  FS-P8: “I mean if you walk downtown like towards the drug area you will see that they have naloxone all over the place...” |
|  |  |  |  |
| What did they think about the study materials and procedures | Experiences of study procedures | Recollections of what it was like to go through the study and its different steps  *Includes*: insights on what worked well or what was lacking in terms of the practical aspects of recruitment, reminders, taking part in the intervention and simulation, what they learned about OEND overall | FS-P1: “it was perfect actually, it was more than what I thought it would be… it was an eye opener… it gave me a good idea of how it, it could be in a situation like that [overdose], so no, it was great.”  FS-34: “So when I found out it [naloxone administration] was nasal I was a lot more relieved. And a lot more welcoming to the idea of doing this course.”  FS-P3: “And you know, with a 5 minute training, I mean realistically it’s, it’s a video and as you have demonstrated, it’s very simple you know. You open it up and it says ‘call 911’ … the packaging is very explanatory you know: shake, shout, call 911…” |
|  | Interactions with the study team | Reflections on how they felt treated in their interactions with research team members | FS-P9: “You guys get it right, like you’re very kind and, and very caring so, so that kind of leaves the door open to come back and, and just keep on with this stuff.” |
|  | Perspectives on the animation | Comments on graphics quality, how information is presented in the animation.  *Includes:* discussion about how the animation helped them understand steps involved; whether they remembered animation | FS**-**P1: “ It got my attention more about the chest compressions, that’s the first time I had ever heard about it, was when you showed me the video [animation].”  FS-P15: “I think it made clear the important steps, and like the real important ones… {Long pause} calling 911, and chest compressions and giving them naloxone and giving it to them again after 5 minutes if they are not breathing. I think it was really good at like getting those important things to stand out.”  FS-P34: “Um, the training video that I’ve seen a week ago, I barely have any recollection of it.” |
|  | Carrying the kit | Experiences carrying kit, reactions to the kit’s design  *Includes:* ease of carrying it; reflections on physical kit design; who you tell you’re carrying it; distributing kits to others | FS-P3: “I have shared it with my friend who was an opioid user, I have shared it with some customers, regulars at work, I have shared it with family.”  FS-P14: “I believe every person that’s using that they should have a kit.” |
